# Prospective preference assessment for the Comparison of Analgesic Regimen Effectiveness and Safety in Surgery (CARES) trial

**DOI:** 10.1186/s13063-022-06123-0

**Published:** 2022-03-04

**Authors:** Brian Hyung, Mark C. Bicket, Richard Brull, Janneth Pazmino-Canizares, Didem Bozak, Karim S. Ladha

**Affiliations:** 1 Temerty Faculty of Medicine, University of Toronto, Toronto, Ontario Canada; 2 Department of Anesthesiology, University of Michigan School of Medicine, Ann Arbor, MI USA; 3 Opioid Prescribing Engagement Network, Institute for Healthcare Policy and Innovation, Ann Arbor, MI USA; 4 Department of Anesthesiology and Pain Management, Department of Anesthesia, Toronto Western Hospital, Toronto, Ontario Canada; 5 Department of Anesthesiology and Pain Medicine, University of Toronto, Toronto, ON Canada; 6 Department of Anesthesia, St. Michael’s Hospital, Toronto, ON Canada; 7 Department of Anesthesiology and Pain Management, Women’s College Hospital, Toronto, Ontario Canada

**Keywords:** Prospective preference assessment, Patient enrollment, Opioids, Pain management

## Abstract

**Background:**

Clinical trials face major barriers such as under-enrollment and selective enrollment, which threaten study completion and undermine validity and generalizability. Thus, we conducted a prospective preference assessment (PPA) prior to commencing the Comparison of Analgesic Regimen Effectiveness and Safety in Surgery (CARES) trial—a randomized controlled study comparing the outcomes of managing acute postoperative pain between opioid-sparing and opioid-based therapies. This PPA aimed to (1) determine the patients’ willingness to participate in the CARES trial, (2) identify the areas for improvement, and (3) assess the differences between willing and unwilling patients.

**Methods:**

Patients undergoing elective laparoscopic cholecystectomy were recruited between August 2019 and February 2020 from two academic hospitals. A survey was administered to each patient consisting of (1) a vignette describing the trial, (2) an assessment of the patients’ understanding of the trial, (3) open-ended questions assessing the attitudes towards the trial, and (4) patient-completed questionnaires. Data were analyzed qualitatively with thematic analysis and quantitatively with the Wilcoxon signed-rank and chi-square tests.

**Results:**

Forty-two patients were enrolled and grouped based on the 6-point Likert scale into willing (4–6, 71%) and not willing (1–3, 29%) to participate in the CARES trial. There were no significant differences with respect to all variables: age, education, sex, visible minority status, previous research, previous surgery, regular use of pain medications, surgical concerns, previous discussions on pain management, significant pain within the past 3 months, and significant use of pain medication within the past month. Factors that motivated participation were contributing to scientific research (45%), altruism (29%), and improving personal pain (24%). Common discouraging factors were negative perceptions of opioids (29%), side effects (21%), being blinded to the study medication (21%), and poor pain management (19%).

**Conclusions:**

This PPA revealed that two key discouraging factors for patients were being blinded to the type of pain medication being taken and the potential for poor pain management as a consequence of participation. Modifications to improve patient acceptance of the CARES trial include ensuring sufficient rescue medicine and follow-up visits consistent with current standards of care for all patients, as well as patient education surrounding safe administration and side effects of the study medications.

**Supplementary Information:**

The online version contains supplementary material available at 10.1186/s13063-022-06123-0.

## Background

While prescription opioids are often assumed to represent the most effective treatment and the standard of care for pain management after discharge from surgery, data to support their superiority over other analgesic medications such as non-steroidal anti-inflammatory drugs (NSAIDs) is lacking. Thus, the balance of benefits versus risks when prescribing opioids for acute surgical pain is currently unclear. We propose to fill this critical gap in evidence by conducting a randomized controlled trial comparing two clinically relevant prescribing strategies—opioid-based (i.e., oxycodone and acetaminophen) or opioid-sparing (i.e., ibuprofen and acetaminophen)—for acute postoperative pain. The Comparison of Analgesic Regimen Effectiveness and Safety in Surgery (CARES) trial will evaluate multiple patient-centered pain and safety outcomes for patients undergoing surgery. Participants will be adults from multiple centers located in Canada and the USA who undergo one of the following outpatient surgical procedures: gallbladder removal, inguinal hernia repair, and breast lumpectomy.

In order to assess the feasibility of the CARES study design, we conducted a prospective preference assessment (PPA) prior to conducting the trial. This is a method first described by Halpern which allows the investigators to assess the views of the proposed study design and garner suggestions of how the study could be made more appealing [1]. The aim of this PPA was to provide a valuable opportunity to evaluate and address the potential concerns that participants may have with the CARES trial, an acute pain study related to opioid administration. Previous studies have reported an increased risk of persistent opioid use after prescription of opioids for acute pain in opioid-naïve patients in the postoperative period [2–5]. These findings, in addition to the increasing awareness of the opioid crisis over the past decade, may challenge patient acceptance of the CARES trial leading to difficulties, particularly with under-enrollment and selective enrollment. Both of these issues are major barriers in conducting a clinical trial and not only threaten the successful completion of a study but also undermine the validity of the obtained results. Based on the results from this PPA study, we sought to identify the areas of improvement in the CARES trial to increase patient enrollment and acceptance of the study.

The objectives of this PPA were to (1) determine the patients’ willingness to participate in the CARES trial, (2) identify the areas for improvement in the trial protocol to better patient enrollment and acceptability, and (3) assess for the differences in characteristics between patients willing to participate and those who would decline the trial.

## Methods

### Setting and population

A purposive sampling framework was used to recruit participants undergoing elective laparoscopic cholecystectomy between August 2019 and February 2020 from the preoperative clinics at two academic hospitals in Toronto, Canada: St. Michael’s Hospital (SMH) and Women’s College Hospital (WCH). Laparoscopic cholecystectomy was selected as the procedure of interest as it is a common procedure with small incisions for which there is uncertainty regarding optimal analgesic management after discharge. The surgical and anesthetic techniques for the procedure are also relatively standardized across practice settings.

To best assess the potential barriers to study participation, the inclusion criteria consisted only of patients undergoing this operation and adults aged 18–70 years old. Patients who did not have a working knowledge of the English language were excluded from the survey. This study was approved by the research ethics board at each participating institution prior to the start of the study, and informed consent was obtained from each patient prior to conducting patient interviews.

### Survey design

A survey was administered to each patient consisting of four sections found in a typical PPA: (1) a brief vignette of the CARES trial, (2) an assessment of the individuals’ understanding of the trial, (3) open-ended questions assessing the attitudes towards the trial, and (4) a structured patient-completed questionnaire. The patient questionnaire included a 6-point Likert scale to quantify the patient’s willingness to participate by asking the question, “How willing would you be to participate in this study?” The possible responses were definitely not (1), probably not (2), maybe not (3), maybe (4), probably (5), and definitely (6). It also consisted of 11 questions to characterize the baseline demographic characteristics, as well as factors which could have potentially influenced the patient’s decision to participate in the trial, including previous surgical experience and regular use of pain medication (see Additional file 1 for the full survey). Surveys were administered to each participant in-person during their preoperative consultation. All interviews were recorded via a tape recorder and transcribed verbatim by two research coordinators.

### Data analysis

The administered survey collected both qualitative and quantitative data. Descriptive statistics were used to examine the distribution of survey responses. The means, standard deviations (SD), and 95% confidence intervals (CI) were computed for continuous variables and percentages for categorical variables. The Wilcoxon signed-rank tests for continuous variables and chi-square tests for categorical variables were employed to detect the significant differences between subjects who were willing and not willing to participate in the trial.

Thematic analysis was used to examine the patterns, codify, and extract meaning from patient interviews to identify motivating and discouraging factors associated with the CARES trial [6]. Initial transcript readings were independently done by two researchers. During readings, preliminary themes were noted and quantified. Similarities and differences between these themes were resolved and classified accordingly by the research group with consensus. Duplications in concepts from and between individual subjects were eliminated. Finally, these concepts were grouped into overarching, independent themes related to the willingness to participate in the CARES trial. Table 1 details the themes and examples of codified phrases belonging to each category.

**Table 1:** Themes and concepts extracted from subject interviews

Themes	Concepts	Example quotes
Altruisim	Desire to help other patients Give back to others	“This study will be helpful for future patients.” “It would benefit people to know which medication work well.”
Scientific advancement	Contribute to research Improve personal education	“I would like to participate to learn more about the medications that help with pain.” “I think research is important.”
Benefit personal pain management	Rescue medications Better pain management due to follow-up	“I think that having this follow up closely, it is kind of nice to have in terms of pain management.” “It would be ok because you will give the back up incase there is much pain.”
Poor pain management	Uncontrolled pain	“I don’t want to suffer.” “Pain is a main concern for me.”
Blinding to medication	Desire for medication preference Not knowing which medication taken	“The concerns will be that I will not know what I am taking.” “I would like to pick what I am using.”
Negative perception of opioids	Addiction Association with illicit activity	“I don’t think I would like to have that drug sold on street.” “Opioids have high addiction properties.”
Side effects	–	“I know Ibuprofen has many side effects like high blood pressure, damage to kidney.” “One concern might be that non-steroidal might upset your stomach.”
Patient co-morbidities	Previous medical conditions	“I have rheumatoid arthritis and I have already an issue with pain and I am medication for that and I have been told not to take Ibuprophen. I have found that Ibuprophen does not work for me anyway.” “I am allergic to one of the medications of the study.”

## Results

### Features of sample population

**Fig. 1:**
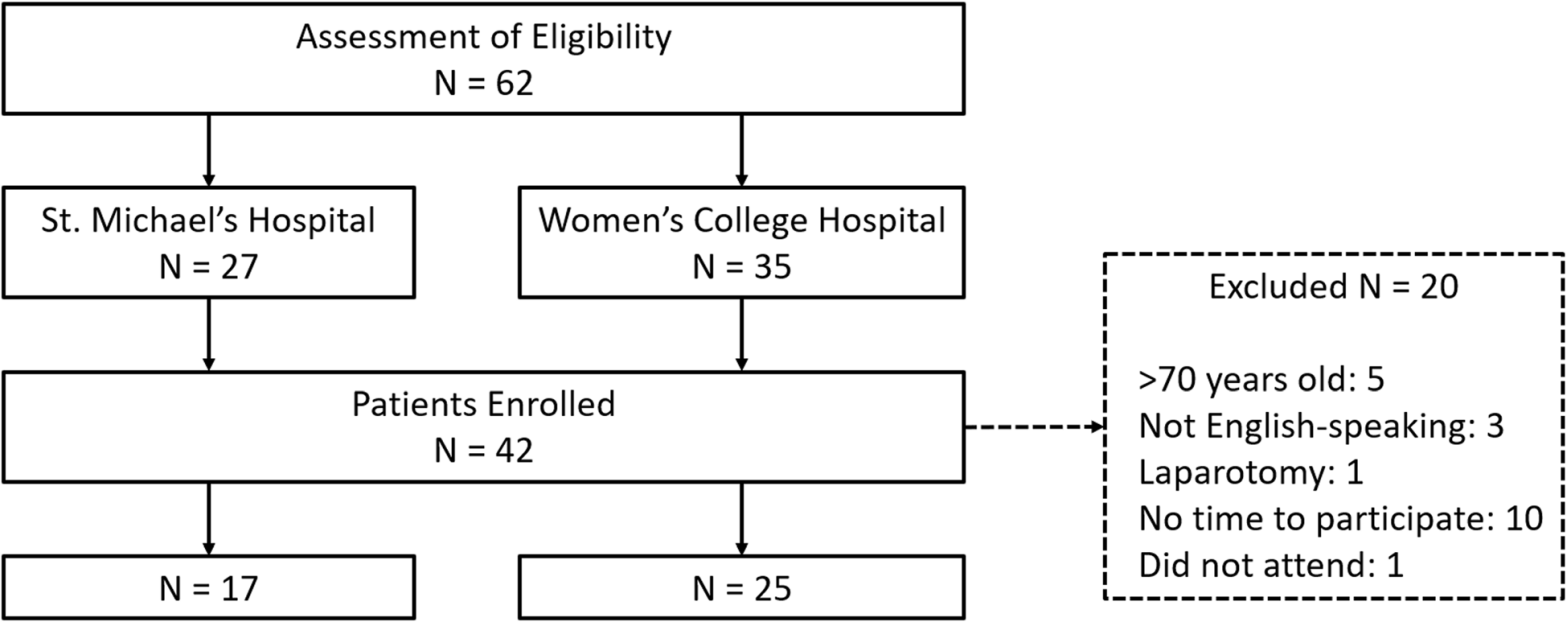
Patient enrollment flowchart

Overall, 62 participants were approached and assessed for eligibility (see Fig. 1). Twenty of these patients did not participate in the study as they did not meet the inclusion criteria (9), did not have time to participate (10), or did not attend (1). Therefore, a total of 42 participants enrolled in this study and completed the survey (SMH 17, WCH 25). Participants were predominantly female (86%) and did not identify as a visible minority (67%). The mean age was 47.6 (SD 14.5), and the mean years of education was 17.3 (SD 2.89). Of the 42 subjects, most were either definitely (17%; CI 6–28%) or probably (43%; CI 28–58%) willing, whereas only a minority of subjects definitely did not (7%; CI 0–15%) or probably did not (14%; CI 4–24%) wish to participate in the CARES trial.

### Patient characteristics influencing willingness

Based on the responses to the 6-point Likert scale, subjects were grouped into willing (4–6; 71%; CI 57–85%) and not willing (1–3; 29%; CI 15–43%) to participate in the CARES trial for the statistical analysis. The Wilcoxon signed-rank tests revealed no statistically significant differences between these two groups with respect to all 11 variables (see Table 2): age (*p* = 0.53), years of education (*p* = 0.22), sex (*p* = 0.09), identification as a visible minority (*p* = 0.47), previous experience with research (*p* = 0.84), previous surgery (*p* = 0.10), use of regular pain medications (*p* = 0.75), concerns with the surgery (*p* = 0.49), previous discussions on pain management (*p* = 0.91), significant pain in the past 3 months (*p* = 0.67), and significant use of pain medication in the past month (*p* = 0.36).

**Table 2:** Association between baseline demographic and clinical characteristics and willingness to participate

Baseline characteristics	Willing (*N* = 30, 71%), *N* (%) or mean ± SD	Not willing (*N* = 12, 29%), *N* (%) or mean ± SD	*p*-value
Age (years)	46.6 ± 14.9	50.1 ± 13.8	0.53
Education (years)	17.7 ± 2.42	16.2 ± 3.71	0.22
Sex (male)	6 (20)	0 (0)	0.09
Visible minority	11 (37)	3 (25)	0.47
Previous research experience	11 (37)	4 (33)	0.84
Previous surgery	20 (67)	11 (92)	0.10
Use of regular pain medications	9 (30)	3 (25)	0.75
Concerns with the surgery	14 (47)	7 (58)	0.49
Previous education on pain management	22 (73)	9 (75)	0.91
Significant pain in the past 3 months	8 (27)	4 (33)	0.67
Significant use of pain medication in the past month	6 (20)	4 (33)	0.36

### Motivating and discouraging factors influencing willingness

A total of 8 independent themes were identified from 42 subject interviews that either motivated (3) or discouraged (5) participation in the CARES trial (see Table 3). Contributing to scientific advancement and research (45%) was the most frequent motivating factor, followed by altruism to other patients (29%) and benefit to personal pain management (24%). Discouraging factors included negative perceptions of opioids (29%), followed by side effects (21%), being blinded to the study medication (21%), potential for poor pain management (19%), and patient co-morbidities (14%).

**Table 3:** Motivating and discouraging factors impacting willingness to participate

Themes	Willing, *N* (%)	Not willing, *N* (%)	Total, *N* (%)
Motivating			
Altruism	11 (26)	1 (3)	12 (29)
Scientific advancement	15 (36)	4 (9)	19 (45)
Benefit personal pain management	9 (21)	1 (3)	10 (24)
Discouraging			
Poor pain management	5 (12)	3 (7)	8 (19)
Blinding to medication	5 (12)	4 (9)	9 (21)
Side effects	5 (12)	4 (9)	9 (21)
Negative perception of opioids	7 (17)	5 (12)	12 (29)
Patient co-morbidities	2 (5)	4 (9)	6 (14)

Of the 41 total responses for motivating factors, a greater proportion were from the willing group (85%) compared to the not willing subjects (15%). Despite this disparity, the same motivating factors were stated by both groups, with no theme unique to either group. Within the theme of benefit to personal pain management, 2 concepts were specific to the CARES trial protocol: the use of rescue medication and scheduled postoperative follow-ups. Three subjects felt the inclusion of follow-up checks was a positive reason to participate in the trial as it contributed to improved pain management. In addition, 5 subjects felt reassured by the use of rescue medication and were more confident they would have adequate pain management if they participated in the trial. In contrast to the motivating factors, the proportion of the 44 total responses for discouraging factors was similar between willing (55%) and not willing (45%) subjects. Participants from both groups stated the same reasons for why they were discouraged from participating in the CARES trial, and there was no one factor that was unique to either group. Within the theme of being blinded to medication, 2 concepts were identified, where participants wished to know which medication would be given to them (7) and desired the option to choose which medication they wanted to take (2).

## Discussion

This PPA study assessed the willingness of individuals to participate in the proposed CARES study, a large randomized controlled trial. Overall, we found that our results reassured the current study design of the CARES trial as the majority of participants, 71%, were willing to participate and only 29% were unwilling. Furthermore, there were no significant differences in the patient characteristics between the two groups to explain the unwillingness to participate.

Previous history with research participation, use of pain medication, and significant pain did not seem to influence whether individuals would participate. However, female sex (*p* = 0.09) and previous surgical history (*p* = 0.10) trended towards not willing to participate. Although a clear association is not established, previous studies by Kerman et al. [7] and Creel et al. [8] have shown that patient treatment preferences are a key factor that influences patient enrollment. For the CARES trial, patients who have had surgery previously may be unwilling to participate as they already have a strong preference towards a therapy that has worked before. With the possibility of not receiving their preferred treatment, there may be less motivation to be involved with the study. This is supported by our qualitative analyses where being blinded to the prescribed pain medication was a commonly cited discouraging factor for subjects. Given this finding, a consideration for the CARES trial would be to modify the study design such that patients are not blinded to the study medication.

Further analysis of patient interviews revealed several factors that may improve patient enrollment and acceptance of the CARES trial. One reason for patients’ unwillingness was the potential for poor pain management by participating in the study. However, patients found that both the use of rescue medication and regular postoperative follow-ups encouraged study participation. While both of these features are already part of the CARES trial, it will be important to ensure sufficient follow-up and rescue medicine consistent with the current standards of care for all patients and to communicate this to all participants during recruitment to reduce apprehension of uncontrolled pain.

Another significant factor that discouraged patient participation was the negative perception of opioids. This wariness is understandable as the opioid crisis has continued to claim lives in the past decades. In 2016 alone, there were 2861 opioid-related deaths in Canada, while this number was 49,860 in the USA in 2019 [9, 10]. Although it is heartening to observe that the awareness of harmful opioid misuse and abuse is increasing, our results suggest that many are wary of properly administered opioids as well. In the context of the CARES trial, it will be important to provide thorough education to patients of the potential side effects of opioids and safe use of these drugs at home to reassure study participants. Furthermore, approaches to address common opioid side effects, such as constipation, pruritus, and nausea, should be discussed with patients during recruitment.

Our findings may be applied broadly when designing or conducting other acute pain studies which may encounter similar discouraging factors for patient participation, including reluctance towards blinding, poor pain management, negative perceptions surrounding appropriate opioid administration, and potential side effects. Consideration of modified study designs such as crossover or unblinded trials with strategies to reduce bias [11] may be potential options to improve enrollment. Furthermore, the utilization of comprehensive patient education, sufficient rescue medication, and follow-ups are crucial aspects that may encourage participation and reassure patients throughout the study duration.

This study was limited by a sample that was skewed towards a population that was well-educated and predominantly female; however, this reflected the demographic characteristics of the local community as well as the gender distribution of gallstone disease [12, 13]. While a small sample population is a limitation, it nonetheless informs our trial. In addition, the skewed demographic in this sample has led our team to identify ways to ensure recruitment across the socioeconomic spectrum. Cholecystectomy is also one of the most common general surgery procedures in Canada and the USA [14, 15], and our results may still be generalized to a large number of patients and similar surgical procedures. We also acknowledge the small sample size. Therefore, we provided 95% CI to demonstrate the precision of our estimates of patient willingness to participate. Lastly, we found that we reached saturation in our interviews of motivating and discouraging factors.

## Conclusions

This study revealed that more than 7 in 10 potentially eligible patients were willing to participate in the CARES trial. While patient demographic characteristics and experience with previous pain, surgery, and use of pain medications did not significantly differ between willing and unwilling subjects, a number of improvable aspects of the CARES trial were identified. Due to the nature of blinded, randomized controlled trials, not all of participants’ discouraging factors may be easily addressed, including blinding to medication and pre-existing patient co-morbidities precluding the use of opioids or NSAIDs. Unfortunately, these factors would not be resolved unless the study design was changed. However, feasible modifications to this trial, which may be generalizable to other acute pain studies, would be ensuring sufficient rescue medication and postoperative follow-up visits, as well as thorough education of safe opioid use during patient recruitment to reduce apprehension surrounding side effects and addiction. Overall, this PPA was able to effectively identify areas of improvement in the CARES trial to facilitate patient enrollment and acceptance.

## Supplementary Information

**Additional file 1.** Patient Survey. Survey administered to each patient consisting of: 1) A vignette describing the trial; 2) An assessment of the patients’ understanding of the trial; 3) Open-ended questions assessing attitudes towards the trial; 4) Patient-completed questionnaires.

**Additional file 2:.** EQUATOR Network Reporting Checklist.

## Data Availability

The datasets used and/or analyzed during the current study are available from the corresponding author on reasonable request.

## Abbreviations

NSAID: Non-steroidal anti-inflammatory drug
CARES: Comparison of Analgesic Regimen Effectiveness and Safety in Surgery
PPA: Prospective preference assessment
SMH: St. Michael’s Hospital
WCH: Women’s College Hospital
SD: Standard deviation
CI: 95% confidence interval
