# Supplementary material for: Prospective preference assessment for the Comparison of Analgesic Regimen Effectiveness and Safety in Surgery (CARES) trial
Source: Trials. 2022 Mar 4;23:195. doi: 10.1186/s13063-022-06123-0 (PMC8895621; doi:10.1186/s13063-022-06123-0)
Supplement: Supplementary file 1 — Additional file 1. Patient Survey. Survey administered to each patient consisting of: 1) A vignette describing the trial; 2) An assessment of the patients’ understanding of the trial; 3) Open-ended questions assessing attitudes towards the trial; 4) Patient-completed questionnaires. [file 13063_2022_6123_MOESM1_ESM.docx]

## **Patient Survey**

*Description of study (to be read to the patient)*

Researchers at St. Michael’s Hospital are studying how to best manage pain after laparoscopic cholecystectomies (removal of the gallbladder). Currently, the best way to manage pain after this type of surgery is not known. One way to treat pain after leaving the hospital is with prescription opioids. Another way to treat pain is with other prescription drugs such as Non-Steroidal Anti-Inflammatory Drugs like Ibuprofen, which may be just as effective at treating pain with fewer side effects. Therefore, we are looking to find patients such as yourself to participate in a study examining the most effective way to treat pain after surgery.

In this study patients will either receive oxycodone or ibuprofen after their surgery to take for pain. The pills will look exactly the same and neither patients nor their doctors will know which pills were given. Which type of pill a patient receives will be random, like flipping a coin. To ensure that pain is treated adequately everyone will also get 5 pills of oxycodone to take as a rescue medication. That means if your pain is not well-treated with the study drug, you can take a rescue pill of oxycodone as needed.

In addition to medication, we will also give participants one of two pamphlets that will provide information about pain after surgery. This will be used to see if education helps with pain management after surgery. Again, which type of pamphlet you receive will be random.

Someone from the research team will call patients at 1 day after their surgery, 3 days after surgery and then at 3 months after surgery to see how well controlled their pain is.

*Questions assessing patients understanding of the trial:*

1. Can you tell me whether patients in the trial will be able to choose or know which medication they are given?
2. Can you tell me what the two types of medications are?
3. Will rescue medication be provided if pain is not well controlled?
4. How will research staff contact you to follow-up with you after the trial?

*Open-ended questions about factors influencing enrollment decisions:*

1. What factors might motivate you to participate in this study?
2. What concerns do you have about participating in this study?

*Questionnaire (to be completed by the patient):*

Considering this information, how willing would you be to participate in this study? Please use the scale from 1 to 6, where 1 means you would definitely not participate, and 6 means you definitely would participate.

| 1 | 2 | 3 | 4 | 5 | 6 |
| --- | --- | --- | --- | --- | --- |
| Definitely Not | Probably Not | Maybe Not | Maybe | Probably | Definitely |

1. What year were you born? _______
2. Sex: Male Female
3. Do you consider yourself to be a visible minority?

Yes No

1. How many years of school have you completed? Please circle one number.

| 1 2 3 4 5 6 7 8 | 9 10 11 12 | 13 14 15 16 | 17 18 19 20+ |
| --- | --- | --- | --- |
| Grade School | High School | Post-Secondary | Graduate School |

1. Have you ever participated in a research study before?

Yes No Not Sure

1. Have you ever had surgery before?

Yes No

1. Are you currently taking any medication for pain regularly (either prescription or over the counter)?

Yes No

1. Is pain one of the major concerns you have regarding your surgery?

Yes No

1. Has anyone discussed how to manage your pain after your surgery?

Yes No

1. Over the past 3 months have you had significant pain on most days?

Yes No

1. Over the past month have you used any medication for pain for more than a week?

Yes No
