# Supplementary material for: Prospective preference assessment for the Comparison of Analgesic Regimen Effectiveness and Safety in Surgery (CARES) trial
Source: Trials. 2022 Mar 4;23:195. doi: 10.1186/s13063-022-06123-0 (PMC8895621; doi:10.1186/s13063-022-06123-0)
Supplement: Supplementary file 2 — Additional file 2:. EQUATOR Network Reporting Checklist. [file 13063_2022_6123_MOESM2_ESM.docx]

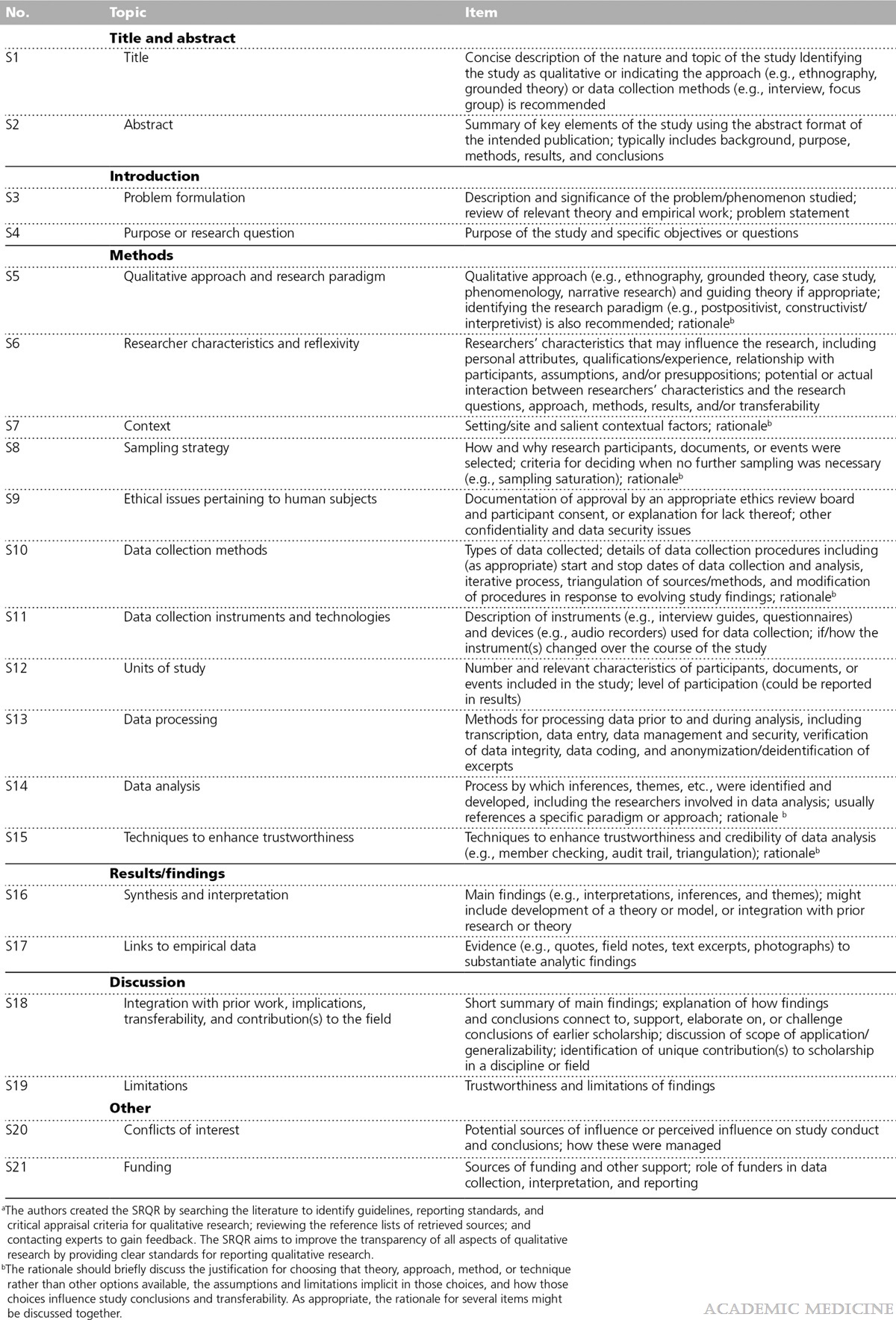


✓

N/A

✓

✓

✓

✓

✓

✓

✓

✓

✓

✓

✓

✓

✓

✓

✓

✓

✓

✓

✓

[**Standards for Reporting Qualitative Research: A Synthesis of Recommendations**](https://journals.lww.com/academicmedicine/Fulltext/2014/09000/Standards_for_Reporting_Qualitative_Research__A.21.aspx)

O’Brien, Bridget C.; Harris, Ilene B.; Beckman, Thomas J.; Reed, Darcy A.; Cook, David A.

Academic Medicine89(9):1245-1251, September 2014.

doi: 10.1097/ACM.0000000000000388
